# Enhanced optical absorption via cation doping hybrid lead iodine perovskites

**DOI:** 10.1038/s41598-017-08215-3

**Published:** 2017-08-10

**Authors:** Zhen-Kun Tang, Zhi-Feng Xu, Deng-Yu Zhang, Shu-Xian Hu, Woon-Ming Lau, Li-Min Liu

**Affiliations:** 1 College of Physics and Electronics Engineering & College of Chemistry and Materials Science, Hengyang Normal University, Hengyang, 421008 China; 2 Beijing Computational Science Research Center, Beijing, 100084 China; 3 Center for Green Innovation, School of Mathematics and Physics, University of Science & Technology Beijing, Beijing, 100083 China

## Abstract

The suitable band structure is vital for perovskite solar cells, which greatly affect the high photoelectric conversion efficiency. Cation substitution is an effective approach to tune the electric structure, carrier concentration, and optical absorption of hybrid lead iodine perovskites. In this work, the electronic structures and optical properties of cation (Bi, Sn, and TI) doped tetragonal formamidinium lead iodine CH(NH_2_)_2_PbI_3_ (FAPbI_3_) are studied by first-principles calculations. For comparison, the cation-doped tetragonal methylammonium lead iodine CH_3_NH_3_PbI_3_ (MAPbI_3_) are also considered. The calculated formation energies reveal that the Sn atom is easier to dope in the tetragonal MAPbI_3_/FAPbI_3_ structure due to the small formation energy of about 0.3 eV. Besides, the band gap of Sn-doped MAPbI_3_/FAPbI_3_ is 1.30/1.40 eV, which is considerably smaller than the un-doped tetragonal MAPbI_3_/FAPbI_3_. More importantly, compare with the un-doped tetragonal MAPbI_3_/FAPbI_3_, the Sn-doped MAPbI_3_ and FAPbI_3_ have the larger optical absorption coefficient and theoretical maximum efficiency, especially for Sn-doped FAPbI_3_. The lower formation energy, suitable band gap and outstanding optical absorption of the Sn-doped FAPbI_3_ make it promising candidates for high-efficient perovskite cells.

## Introduction

Over the last several years, hybrid organic-inorganic perovskite solar cells have become one of the most attractive photovoltaic technologies, with easy solution fabrication and high conversion efficiencies^1–8^. The first perovskite based solar cells, made seven years ago by Japanese researchers, turned just 3.8% of the energy in sunlight into electricity^9^. After that, the efficiency of perovskite solar cells has been updated rapidly as a result of new strategies adopted in their fabrication process^10–18^, including device structure, interfacial engineering, chemical compositional tuning, and crystallization kinetics control. The power conversion efficiency of perovskite solar cells can greater than 20%^18, 19^, which is comparable to the commercial silicon (20%), CIGS (19.6%), GaAs (18.4%) and CdTe (19.6%) solar cells^20^. More recently, a power conversion efficiency up to 22.1% under the operational condition is achieved^21^. The power conversion efficiency of perovskite solar cells is climbing faster than that of any solar technology before them.

The outstanding light absorption is one of the indispensable conditions for the high efficiency solar cells. While, the band gap plays a vital role of light absorption. If the band gap is too small, the device will be able to collect extra current but the open-circuit voltage will be too small. However, if the band gap is too wide (>2 eV), only a small fraction of solar energy can be absorbed. Thus, an absorbing layer with a band gap of approximately 1.4–1.6 eV is preferred for solar cells developed from a single junction^22^. Perovskite materials are built by the inorganic elements lead and iodine, together with simple organic compounds. Most of previous works are mainly focused on the methylammonium lead iodide (MAPbI_3_) perovskites, with a band gap of ∼1.55 eV^23–29^. Compared with un-doped MAPbI_3_ perovskite, Sn-doped MAPbI_3_ perovskite have a small band gap, which can further enhance the photovoltaic performance of perovskite solar cells in the near-infrared spectrum^30, 31^. Besides, Sn-doped MAPbI_3_ perovskite allowed tunable band gap of the perovskite absorber by varying the Sn:Pb ratio^32, 33^.

Once replacing organic compound methylammonium (MA) with formamidinium (FA), a slightly larger organic molecule, the absorption spectrum of perovskite is mostly concentrated in the visible and near-infrared regime^34–36^. Especially for the tetragonal FAPbI_3_ perovskite with a band gap of 1.43 eV, which is therefore potentially superior than the trigonal FAPbI_3_ as the light harvester^35^. Besides, the FA induced structural variability improved charge transport and red-shifted absorption in tetragonal FAPbI_3_ structures^36^. More importantly, the highest confirmed record power conversion efficiency of PSCs is based FAPbI_3_ perovskite^18^. We note that the FASnI_3_ has a band gap of 1.41 eV which allows light harvesting from the near-infrared region^37^. Thus, we very curious to know the electronic structures and optical absorption properties of Sn and other cation-doped tetragonal FAPbI_3_ perovskite.

In this work, first-principles calculations were carried to systematically examine the geometry, electronic structure, and optical properties of the cation (Bi, Sn, and TI) doped tetragonal MAPbI_3_/FAPbI_3_ perovskites. The formation energies and detailed defect-I bond lengths of cation-doped MAPbI_3_/FAPbI_3_ are showed in the Table 1. The calculated results show that the Sn-doped defect is the common impurity in the tetragonal MAPbI_3_/FAPbI_3_ perovskites due to the lowest formation energy of about 0.3 eV. While, relatively higher formation energy means that both the donor defect Bi and the acceptor defect TI are difficult to dope in MAPbI_3_/FAPbI_3_ perovskites. The calculated band gap of Sn-doped MAPbI_3_/FAPbI_3_ perovskite is 1.30/1.40 eV, respectively. The band gap of Sn-doped MAPbI_3_/FAPbI_3_ perovskite is smaller than that of un-doped perovskites. More importantly, the Sn-doped MAPbI_3_ and FAPbI_3_ perovskites have the higher specific absorption in the visible light region, especially for the Sn-doped tetragonal FAPbI_3_ perovskite. Our electronic structures and optical properties calculations indicate that the Sn-doped tetragonal FAPbI_3_ is a promising candidate for high-efficient perovskite cell.

**Table 1:** The formation energies and detailed defect-I bond lengths of cation-doped MAPbI_3_/FAPbI_3_.

Structure	E_f_ (eV)	L-H_defect-I_ (Å)	L-V_defect-I_ (Å)
Bi-doped MAPbI_3_	1.31	3.10	3.29
Sn-doped MAPbI_3_	0.28	3.13	3.21
TI-doped MAPbI_3_	0.88	3.19	3.28
Bi-doped FAPbI_3_	1.36	3.21	3.12
Sn-doped FAPbI_3_	0.29	3.23	3.12
TI-doped FAPbI_3_	0.84	3.33	3.16

Where the E_f_, L-H_defect-I_, and L-V_defect-I_ are the formation energies, the average defect-I bond lengths in the horizontal surface of defect-I octahedral structure and the average defect-I bond lengths in the vertical direction of defect-I octahedral structure, respectively.

## Results

**Figure 1:**
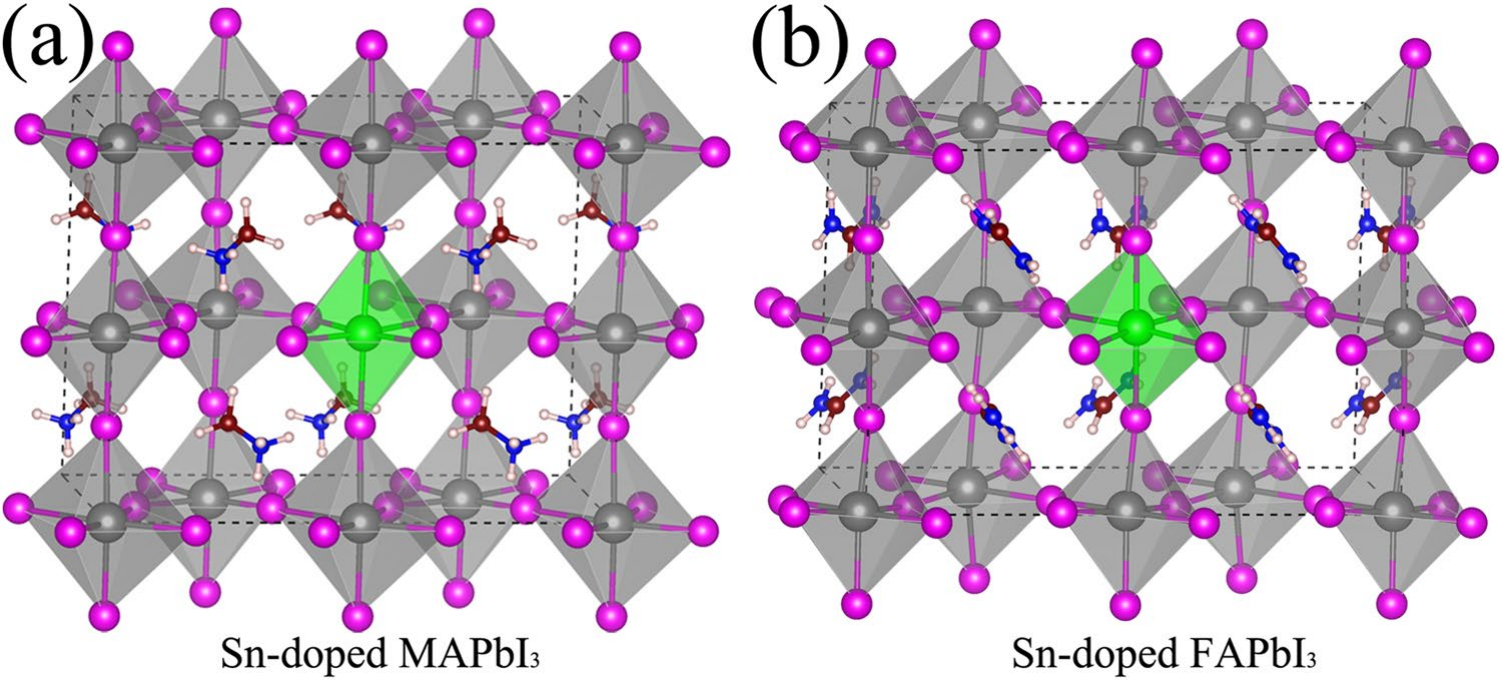
The relaxed atomic structures of the (**a**) Sn-doped MAPbI_3_ and (**b**) Sn-doped FAPbI_3_. The silver, violet, blue, brown, pink, and green balls represent Pb, I, N, C, H, and Sn atoms, respectively.

Before the optimization of the cation-doped perovskite structure, the lattice constants of tetragonal MAPbI_3_ and FAPbI_3_ supercells are fully relaxed. In the tetragonal MAPbI_3_ supercell, the relaxed lattice constants *a* is 8.72 Å and *c* is 12.92 Å, which is in good agreement with the experimental results^32^. In addition, the relaxed lattice constants *a* is 9.20 Å and *c* is 12.54 Å in the tetragonal FAPbI_3_ supercell. Then, we fix the lattice constants in the structural optimization of the cation-doped MAPbI_3_/FAPbI_3_ supercells. Considered the ion radius and the number of outside electrons, three types of atoms (Bi, Sn, and TI) were chosen as the typical cation-doped in the MAPbI_3_ and FAPbI_3_. The Bi and TI represent the donor impurity and acceptor impurity, respectively. The outsider electron number of Pb equal to the Sn atom, which is neither an acceptor impurity, nor a donor impurity. The relaxed structures of Sn doped MAPbI_3_ and FAPbI_3_ supercells are showed in Fig. 1(a) and (b). For the Sn-I octahedral structure in the Sn doped MAPbI_3_, the average horizontal and vertical Sn-I bond length is 3.13 Å and 3.21 Å, respectively. The Sn-I octahedral structure in the Sn doped MAPbI_3_ is a tensile octahedron, which vertical Sn-I bond length larger than the horizontal Sn-I bond length. However, the vertical and horizontal Sn-I bond length is 3.23 Å and 3.12 Å in the Sn doped FAPbI_3_. It means that the Sn-I octahedral structure in the Sn doped FAPbI_3_ is a compressed octahedron.

**Figure 2:**
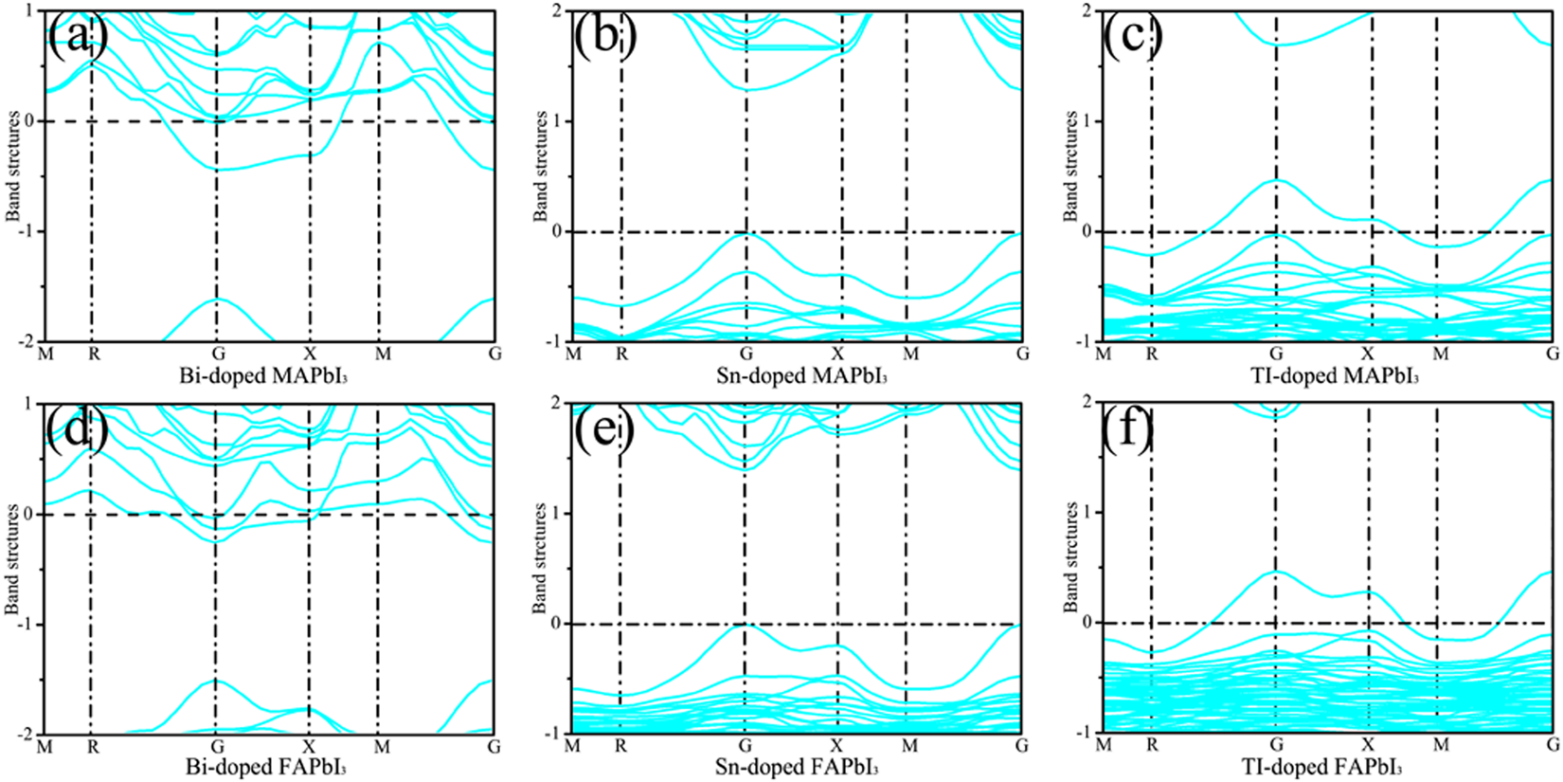
The band structures of the (**a**) Bi-doped MAPbI_3_, (**b**) Sn-doped MAPbI_3_, (**c**) TI-doped MAPbI_3_, (**d**) Bi-doped FAPbI_3_, (**e**) Sn-doped FAPbI_3_, (**f**) TI-doped FAPbI_3_.

Previous experimental and theoretical research^25^ shows that the band gaps and the optical properties of MAPbI_3_ are influenced by the cation-doping. Interesting, the Sn-doped MAPbI_3_ possesses a favorable band gap and even greater optical absorption in the visible-light region. Therefore, it is necessary to know whether the cation-doped FAPbI_3_ has fascinating electronic and optical properties. The band structures of the cation-doped MAPbI_3_ and FAPbI_3_ are calculated by density functional theory (DFT) with PBE functional. The PBE functional calculations can give reasonable electronic properties of hybrid lead iodine perovskite structure^38^. In the Bi-doped MAPbI_3_/FAPbI_3_, the Fermi level across the conduction band due to the donor defect Bi, as shown in the Fig. 2(a) and (d). While, the Sn-doped MAPbI_3_/FAPbI_3_ is the perfect semiconductor with a direct band gap at the G point. The calculated band gap of Sn-doped MAPbI_3_ and FAPbI_3_ is 1.30 eV and 1.40 eV, respectively. Compare with the band gap of un-doped tetragonal MAPbI_3_ (1.50 eV) and FAPbI_3_ (1.57 eV), the Sn-doped MAPbI_3_ and FAPbI_3_ have the relatively lower band gap for broader-spectrum light harvesting. For the TI-doped MAPbI_3_/FAPbI_3_ structure, the Fermi level is lower than the valence band maximum, as shown in the Fig. 2(c) and (f). Thus, the TI is a shallow acceptor defect in the TI-doped MAPbI_3_/FAPbI_3_ structure.

**Figure 3:**
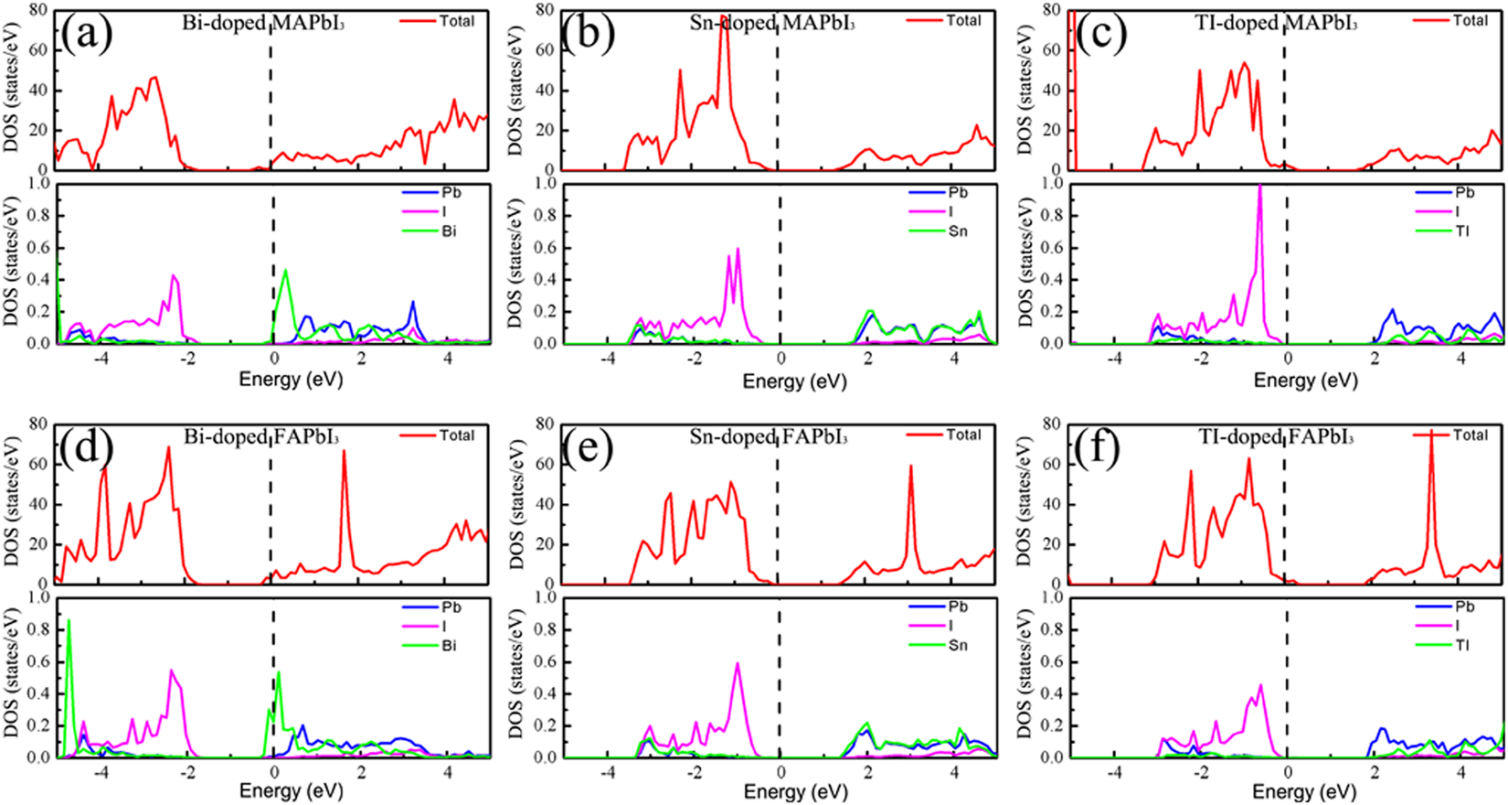
The total DOS and PDOS of Pb, I, and defect in the (**a**) Bi-doped MAPbI_3_, (**b**) Sn-doped MAPbI_3_, (**c**) TI-doped MAPbI_3_, (**d**) Bi-doped FAPbI_3_, (**e**) Sn-doped FAPbI_3_, and (**f**) TI-doped FAPbI_3_. In order to facilitate comparison with PDOS of defect, the PDOS of Pb and I is the average PDOS per atom. The total DOS and PDOS are shown on the upper and low panel in the subfigure. The red, blue, violet, and green lines represent the total DOS, PDOS of Pb, I, and doped cation, respectively.

To get a deeper understanding the electronic properties of the cation-doped MAPbI_3_/FAPbI_3_, the total density of states (DOS) and partial density of states (PDOS) of Pb, I and cation defects are plotted in the Fig. 3. The partial DOS of Bi-doped MAPbI_3_/FAPbI_3_ shown that the electronic states near the Fermi level are mainly contributed by Bi defects, as shown in the Fig. 3(a) and (d). For the Sn-doped MAPbI_3_/FAPbI_3_, the valence band maximum (VBM) is mainly contributed by I atom, while the conduction band minimum (CBM) is mainly contributed by Sn and Pb atom. Besides, most PDOS of Sn defect are overlapped with the PDOS of single Pb atom becuase Sn and Pb have the same outer electron configuration. Compared with the PDOS of Pb atom, more sattes of TI in the TI-doped MAPbI_3_/FAPbI_3_ distributed in the high energy region, which further confirms that the TI is an acceptor defect in the doped MAPbI_3_/FAPbI_3_ systems.

**Figure 4:**
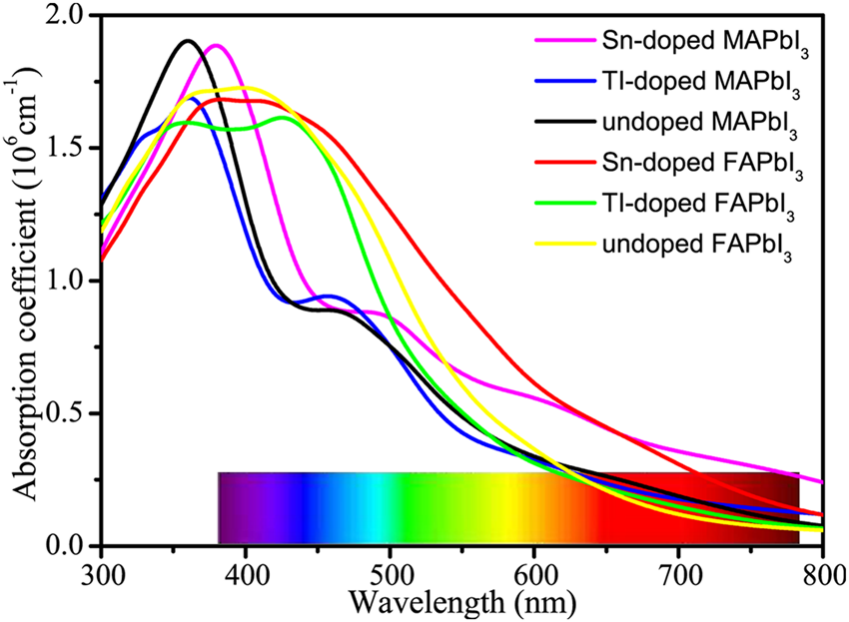
The calculated optical absorption spectra of the doped and undoped MA(FA)PbI_3_. The violet, blue, black, red, green, and yellow lines represent the optical absorption spectra of the Sn-doped MAPbI_3_, TI-doped MAPbI_3_, un-doped MAPbI_3_, Sn-doped FAPbI_3_, TI-doped MAPbI_3_ and un-doped MAPbI_3_, respectively.

To evaluate the optical absorption of halide perovskites, the optical absorption efficients of the Sn and TI doped MAPbI_3_/FAPbI_3_ perovskites are calculated and compared with the un-doped MAPbI_3_/FAPbI_3_ perovskites, as shown in Fig. 4. For the Sn-doped MAPbI_3_/FAPbI_3_, the optical absorption peak is lower than that of undoped MAPbI_3_/FAPbI_3_. However, the Sn-doped MAPbI_3_ has better light absorption in the visible regions (380–780 nm), which is consistent with recent theoretical and experimental results. In contrast to the un-doped MAPbI_3_/FAPbI_3_, the optical absorption peak of Sn-doped MAPbI_3_/FAPbI_3_ exhibits a red-shift. But the optical absorption spectrum of TI-doped MAPbI_3_/FAPbI_3_ is lower than that of un-doped structures in most of the visible light region. At the strongest emission ares of sunlight (450–500 nm), the absorption efficient of Sn-doped FAPbI_3_ is about in 1.5 × 10^6^ cm^−1^, which is 1.5 times larger than that of Sn-doped MAPbI_3_. Considering the range of visible light accounts for the major usable portion of the full solar spectrum, the visible light absorption is critical to achieve high efficiency cells. Therefore, it is very essential to know whether the Sn-doped FAPbI_3_ can enhance the photoelectric conversion efficiency.

**Figure 5:**
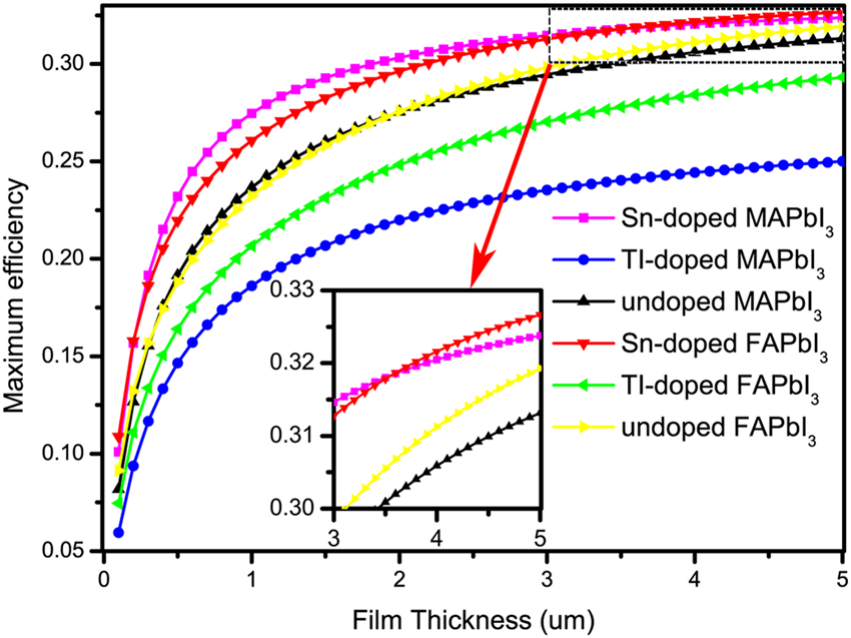
The calculated maximum efficiencies of the cation-doped and undoped MA(FA)PbI_3_. The violet, blue, black, red, green, and yellow lines represent the maximum efficiencies of the Sn-doped MAPbI_3_, TI-doped MAPbI_3_, un-doped MAPbI_3_, Sn-doped FAPbI_3_, TI-doped MAPbI_3_ and un-doped MAPbI_3_, respectively.

In general, the effect of the optical absorption coefficient is not considered in the well-known Shockley-Queisser limit^39^. The theoretical maximum efficiency depends on the thickness of the absorber layer^40–42^. Yin *et al*.^26, 43^ calculated the thickness-dependent maximum solar cell parameters of CH_3_NH_3_PbI_3_ based on Fermi Golden rule. According to the Fermi Golden rule, the optical absorption of a photonic energy ħ*ω* is directly correlated with $\frac{2{\rm{\pi }}}{{\rm{\hslash }}}\int |  < v| \hat{H}{| c > | }^{2}\frac{2}{8{\pi }^{3}}\delta ({E}_{c}(\vec{k})-{E}_{v}(\vec{k})-{\rm{\hslash }}{\rm{\omega }}){d}^{3}k$, Where $< v| \hat{H}| c >$ is the transition matrix from states in the valence band (VB) to states in the conduction band (CB) and the integration is over the whole reciprocal space. For a real solar cell, the theoretical maximum efficiency depends on the thickness of the absorber layer^43^. After taking the absorption efficient and absorber layer thickness into consideration, we have calculated the maximum efficiencies of some common light absorbers as a function of the thickness of the absorber layers, as shown in Fig. 5. With a 5 µm absorber, the maximum efficiency of Sn-doped MAPbI_3_, TI-doped MAPbI_3_, un-doped MAPbI_3_, Sn-doped FAPbI_3_, TI-doped FAPbI_3_, and un-doped FAPbI_3_ based cells is 32.4%, 25.0%, 31.3%, 32.7%, 29.3%, and 31.9%, respectively. Obviously, the Sn-doped MAPbI_3_/FAPbI_3_ perovskites exhibit much higher conversion efficiencies than un-doped MAPbI_3_/FAPbI_3_ and TI-doped MAPbI_3_/FAPbI_3_ for any given thickness. More importantly, the Sn-doped MAPbI_3_/FAPbI_3_ perovskites are capable of achieving high efficiencies with very thin absorber layers. For example, with a 0.5 µm absorber, Sn-doped MAPbI_3_ and Sn-doped FAPbI_3_ based cells can have a maximum efficiency up to 23.2% and 21.9%, respectively. Considering the strong capacity of light absorption and high maximum efficiency, the Sn-doped tetragonal FAPbI_3_ should be a more suitable candidate for the high efficiency perovskite solar cell material.

## Discussions

To know the difficulty of Bi, Sn, and TI doping in the MAPbI_3_ and FAPbI_3_, the formation energy, E_f_, is calculated. The formation energy of the cation-doped MAPbI_3_/FAPbI_3_ is defined as follows,

1 $${E}_{f}={E}_{total}\,-\,{E}_{pure}+{\mu }_{Pb}-{\mu }_{cation}$$

where *E*_*total*_, *E*_*pure*_, *μ*_*pb*_ and *μ*_*cation*_ represent the total energy of the cation-doped MAPbI_3_/FAPbI_3_, the total energy of the primitive MAPbI_3_/FAPbI_3_, the chemical potential of Pb atom, and the chemical potentials of doped cations, respectively. In our calculations, the chemical potentials of the Pb and doped cations use the formation enthalpy of the corresponding most stable metal structures. The calculated formation energies are 1.31, 0.28, 0.88, 1.36, 0.29 and 0.84 eV for the Bi-doped MAPbI_3_, Sn-doped MAPbI_3_, TI-doped MAPbI_3_, Bi-doped FAPbI_3_, Sn-doped FAPbI_3_, and TI-doped FAPbI_3_ supercell, respectively. The relatively small formation energy of Sn-doped MAPbI_3_/FAPbI_3_ indicates that it is easy to dope Sn in the tetragonal MAPbI_3_/FAPbI_3_ structure. The formation energy results indicate that n-type MAPbI_3_/FAPbI_3_ halide perovskites (Bi-doped) are more difficult to form than p-type MAPbI_3_/FAPbI_3_ halide perovskites (TI-doped).

In this work, the electronic structures and optical properties of typical cation (Bi, Sn, and TI) doped MAPbI_3_/FAPbI_3_ are studied by density functional theory. The calculation results show that both the donor defect Bi and the acceptor defect TI have the relatively high formation energies. While, the Sn defect is easy to dope in the tetragonal MAPbI_3_/FAPbI_3_ structure due to the small formation energy of 0.3 eV. The calculated band gap of Sn-doped MAPbI_3_ and FAPbI_3_ is 1.30 eV and 1.40 eV, respectively. The optical absorption efficients of Sn-doped MAPbI_3_/FAPbI_3_ are higher than that of un-doped MAPbI_3_/FAPbI_3_ within the visible light range. More importantly, the Sn-doped MAPbI_3_/FAPbI_3_ have relatively high theoretical maximum efficiency, especially for the Sn-doped FAPbI_3_. The lower formation energy, suitable band gap and outstanding optical absorption of the Sn-doped FAPbI_3_, enable it has great potential applications for the high-efficient perovskite cells.

## Method

The first-principles structure, energy and optical absorption calculations were performed by the Vienna Ab Initio Simulation Package (VASP)^44, 45^. Projector augmented-wave (PAW) pseudopotentials^46^ were used to account electron-ion interactions. The generalized gradient approximation (GGA) with the PBE functional^47^ was used to treat the exchange-correlation interaction between electrons. In order to get the appropriate doping concentration, 2 × 1 × 1 MAPbI_3_ and FAPbI_3_ supercells are used in our calculation. The energy cutoff was set to 500 eV and a 5 × 7 × 7 Monkhorst-Pack scheme was used to sample Brillouin zone^48^. The full geometry optimizations are carried out with the convergence thresholds of 10^−4^ eV and 1 × 10^−2^ eV/Å for total energy and ionic force, respectively. It is well-known that vdW interactions are crucial in the determination of the equilibrium configurations in the hybrid structure. Thus, the DFT-D3 approach was used to take the effect of the vdW interaction^49^.

It is well known that the PBE functional always underestimated the band gap of semiconductors. Besides, the spin-orbit coupling (SOC) also results in much reduced band gaps in hybrid lead iodine perovskite structure. In the previous DFT calculation, both the hybrid HSE06 functional and spin-orbit coupling effects are considered to calculated the electronic properties of hybrid lead iodine perovskite structure. Their calculated results show that the band gap of cubic MAPbI_3_ is 1.60 eV with PBE functional, while the band gap of PBE + SOC and PBE + HSE + SOC is 0.49 eV and 1.53 eV^38^. It is noted that the band gaps obtained by PBE without including SOC is quite close experimental value of 1.55 eV^9^. Thus, the PBE functional could give the reasonable band gaps for hybrid lead iodine perovskites. In addition, the calculated optical of cation-doped MAPbI_3_/FAPbI_3_ with PBE functional also should show the right trend.
